# Cumulative live birth rate according to the number of receiving governmental subsidies for assisted reproductive technology in Saitama Prefecture, Japan: A retrospective study using individual data for governmental subsidies

**DOI:** 10.1002/rmb2.12397

**Published:** 2021-06-20

**Authors:** Seung Chik Jwa, Osamu Ishihara, Akira Kuwahara, Kazuki Saito, Hidekazu Saito, Yukihiro Terada, Yasuki Kobayashi, Eri Maeda

**Affiliations:** ^1^ Department of Obstetrics and Gynecology Saitama Medical University Saitama Japan; ^2^ Department of Obstetrics and Gynecology Graduate School of Biomedical Sciences Tokushima University Tokushima Japan; ^3^ Department of Comprehensive Reproductive Medicine Graduate School Tokyo Medical and Dental University Tokyo Japan; ^4^ Umegaoka Women's Clinic Tokyo Japan; ^5^ Department of Obstetrics and Gynecology Graduate School of Medicine Akita University Akita Japan; ^6^ Department of Public Health Graduate School of Medicine The University of Tokyo Tokyo Japan; ^7^ Department of Environmental Health Science and Public Health Akita University Graduate School of Medicine Akita Japan

**Keywords:** assisted reproductive technology, embryo transfer, intracytoplasmic sperm injection, in‐vitro fertilization, pregnancy rate

## Abstract

**Purpose:**

We investigated the cumulative live birth rate (CLBR) in women receiving governmental subsidies for assisted reproductive technology (ART) in Saitama Prefecture, Japan.

**Methods:**

Women who applied for subsidies from Saitama Prefectural Government for the first time in 2016 were enrolled and followed up until the end of 2017. Treatment information, including live birth, was obtained from the Japanese ART registry by linking it with unique identification numbers for treatment. Patients’ factors associated with having a live birth were investigated.

**Results:**

Of 1,072 women (2,513 applications), 495 (46.2%) had a live birth with 8 (1.6%) twin pregnancies. The CLBR over six subsidized cycles was 53.7% for women aged <40 years, and 17.2% over three subsidized cycles for women 40‐42 years; highest among women <35 years (58.4%), followed by those aged 35‐39 years (49.3%). Multivariate analysis revealed patient age as the only independent factor for having a live birth.

**Conclusions:**

The CLBR of women receiving subsidies for ART was greatest in women aged <35 years. Effective policies for promoting ART among younger couples who seek infertility treatment are essential.

## Graphical abstract

The overall cumulative live birth rate across six sets of subsidies for women aged <40 years was 53.7%; highest among women aged <35 years (58.4%), followed by those aged 35‐39 years (49.3%). For women aged 40‐42 years who were allowed three sets of subsidies, the CLBR was 17.2%. Multivariate analysis revealed only patient age as an independent factor associated with having a live birth.

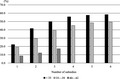

## 1 INTRODUCTION

Assisted reproductive technology (ART) is now a widely accepted infertility treatment in Japan. According to the latest report from the Japan Society for Obstetrics and Gynecology (JSOG), 454,893 treatment cycles were implemented and 56 979 neonates were born during 2018, both of which are increased from previous years and are the highest since the registry was launched in 2007^1^ despite a rapidly decreasing trend of the total numbers of babies born in Japan.^2^ One of the main reasons for the increased number of ART cycles in Japan is attributable to the advanced age for women seeking this option. As is common in many developed countries,^3^ women tend to delay child bearing in Japan,^4^ and face an increased risk of infertility related to advanced age; from the report of the JSOG, 41.8% of registered ART cycles were those for women in their 40s,^1^ which is extremely high compared with other countries.^5^

Although the health insurance system in Japan provides universal coverage,^6^ ART treatment is an exception, and patients must pay for ART treatment out of their own pockets. However, the Japanese government currently reimburses a part of the ART treatment cost for six attempts for women aged <40 years, and for three attempts for women aged 40‐42 years since 2014. There was an upper limit of 7 300 000 JPY (approximately 70 200 USD using a 2021 exchange rate of 1 USD = 104 JPY) per annual couple's income for receiving the subsidies, but this policy was modified in January 2021 because of the recent stagnation in the Japanese total fertility rate.^7^ The newly introduced elimination of the income limit is expected to further increase the number of ART treatment cycles. However, to date, the live birth impact of governmental subsidies for ART treatment has not been fully evaluated.^8^ More specifically, among those receiving subsidies, little is known about who had a live birth in terms of age groups, infertility reasons, income levels, or the number of subsidized cycles. Such evaluation is essential for drafting an effective policy for the utilization of ART treatment in Japan. However, in Japan, nationwide individual‐based data on the governmental subsidies for ART is not available, partly because the Ministry of Health Labour and Welfare has not collected such data and partly because the governmental subsidies were mainly managed by local municipalities including a core city (populations >200 000), an ordinance‐designated city, and otherwise prefectural government. Therefore, we aim to investigate CLBR according to the number of receiving governmental subsidies in women stratified by different age groups using individual data managed by Saitama Prefectural Government.

## 2 MATERIALS AND METHODS

This is a retrospective study using individual data based on the application of governmental subsidies for ART managed by Saitama Prefectural Government. We applied for access to individual data for ART subsidies in anonymous form to Saitama Prefectural Government during 2016 and 2017. In Saitama prefecture, governmental subsidies were managed by core cities with populations >200 000 (Koshigaya, Kawagoe, and Kawaguchi), and an ordinance‐designated city (Saitama city) and otherwise Saitama Prefectural Government, and the same applied to the individual data related to them. The population and number of households covered by this study in 2016 were 5 315 797 and 2 978 871, respectively: being 73.1% and 72.5% of the overall Saitama Prefecture because the above cities were excluded. Kawaguchi was designated as a core city in 2018, and applicants from the city before 2018 were included in this study. Subsidies for ART are eligible for couples in which the woman is aged less than 43 years and with an annual couple's income of less than 7 300 000 JPY. This study was approved by the institutional review board of Saitama Medical University (Approval number, 904; September 2019) and the ethics committee of the JSOG (Approval number 2020‐2; June 2020). After these approvals, the Saitama Prefectural Government provided us with access to data without any identifying information.

Individual data for application used for the analysis included the number of applications, patients’ age, and the husband's and wife's annual income. Treatment types were classified as follows: A, cycles with fresh embryo transfer (ET); B, Cycle with freeze‐all and a subsequent frozen‐thawed embryo transfer (FET); C, FET cycles; D, cancelation because of patients’ health problems; E, cancelation because of failed fertilization or embryo development; and F, cancelation because of failed oocyte collection. We also used the fertilization methods for fresh ET cycles including in vitro fertilization (IVF), intracytoplasmic sperm injection (ICSI), and the results in terms of conception from the application information. Husband's and wife's annual income was based on a proof of earnings for prefectural tax or a taxation certificate. Women who reported no income were defined as housewives. The data also included unique identification numbers for the designated treatment cycle based on the Japanese ART registry in which almost all treatment cycles conducted in Japan are registered. This study linked the individual data for application and the Japanese ART registry to confirm a live birth status. From the Japanese ART registry, information on infertility diagnosis, fresh/FET status, ovarian stimulation protocols for fresh cycles, fertilization methods (IVF/ICSI), number of collected oocytes, number of embryos transferred, and number of embryos cryopreserved and pregnancy outcomes including live birth status was used for the analysis.

**FIGURE 1:**
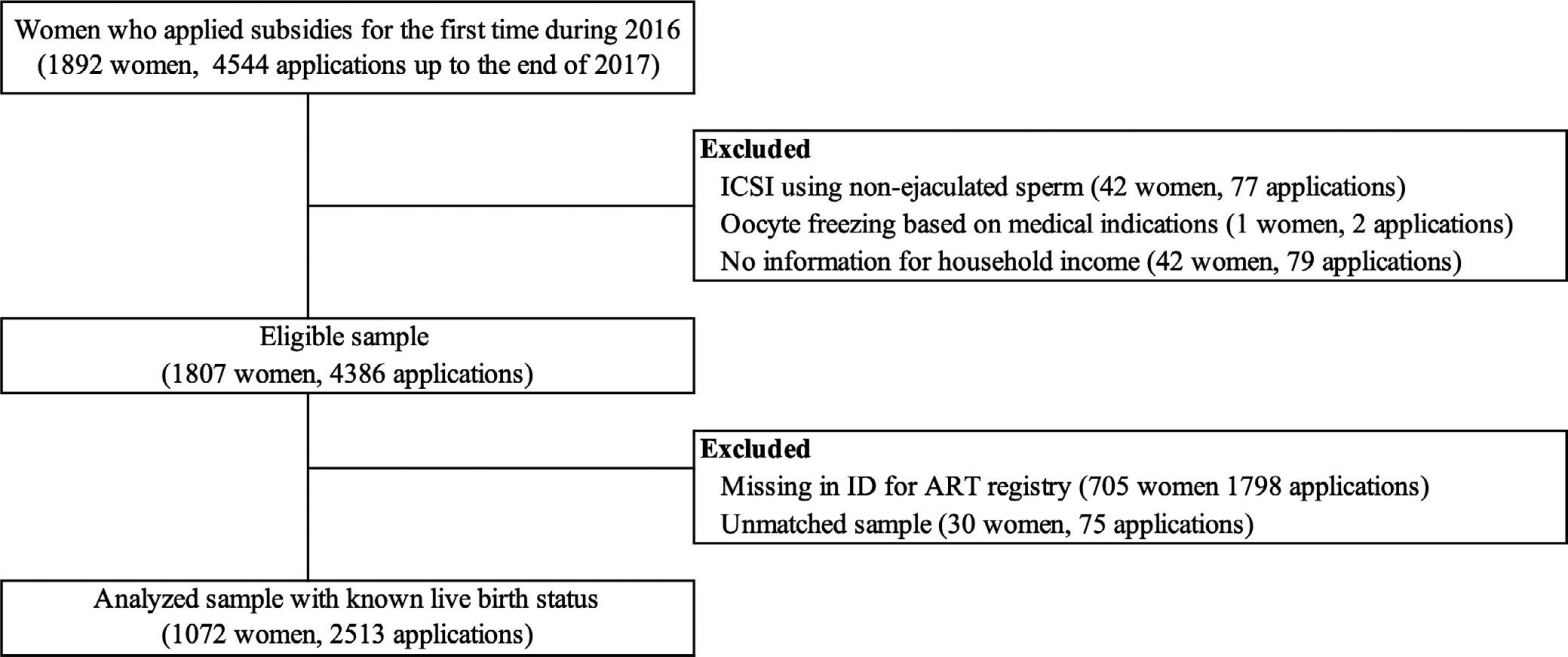
Flow diagram of sample selection

A flow diagram of this study is shown in Figure 1. In all, 1872 women applied governmental ART subsidies for the first time during 2016. Among these, ICSI using non‐ejaculated (testicular or epididymal) spermatozoa, and cycles with oocyte freezing based on medical indications, or with no information for couple's income were excluded. Of the remaining 1807 women considered as eligible for sampling, 705 women (39.0%) were excluded because of missing identification numbers in the ART registry in at least one of the applications, and 30 women were excluded as they were unmatched in the ART registry. Finally, 1072 women and 2513 applications up to the end of 2017 with live birth status were included for the analysis.

Using these data, we have calculated the cumulative live birth rate (CLBR) according to the number of applications for subsidies. The definition of CLBR was the first live birth per individual patient. We took a conservative approach to calculate this, assuming that none of the women who discontinued making applications would have had a live birth. Thus, the numerator was the number of live births divided by the total number of women included. Once a woman had achieved her first liveborn baby, they did not contribute to any further CLBRs for our analysis. CLBRs were calculated for three age groups based on the first application for a subsidy in 2016 (<35, 35‐39, and 40‐42 years). For women aged 40‐42, governmental subsides were only allowed for three ART cycles, and CLBRs were calculated up to this level for all.

### 2.1 Statistical analysis

First, application information was analyzed for the included and excluded samples among the eligible sample (n = 1807) to evaluate the pattern of inclusion. Then, baseline characteristics were analyzed according to different age groups using chi‐squared or Fisher's exact tests or one‐way analysis of variance as appropriate. Among the analyzed sample, information on fresh cycles at the first application was available for 980 women, and the treatment information for fresh cycles was analyzed according to the different age groups. Finally, patient factors associated with having a live birth were investigated. Because the outcomes were not rare (ie, >10%), we directly estimated relative risks (RRs) of patient factors for having a live birth using a generalized linear model for a binomial family with log‐link function instead of calculating odds ratios using logistic regression modeling.^9^ In the multivariable model, age at the time of first application in 2016, infertility diagnosis, quartiles of annual couple's income, and housewife status were included. All analyses were conducted using the STATA MP statistical package, version 16.0 (Stata). A two‐tailed *P* value of <.05 was considered statistically significant.

## 3 RESULTS

Characteristics based on application information for the eligible sample of women stratified according to inclusion/exclusion status are shown in Table 1. The included women were more likely to include cycles with fresh ET compared with excluded women at the first application, while the proportions of freeze‐all and a subsequent FET and canceled cycles were higher among the excluded women, with statistical significance (*P* < .001). The mean total number of subsidies and mean annual couple's income were significantly higher among the excluded women (2.34 [standard deviation, SD ± 1.28] for analyzed sample vs. 2.55 [SD ± 1.44] for excluded sample, *P* = .002).

**TABLE 1:** ART cycle characteristics based on application information for eligible samples stratified by included and excluded status (n = 1807)^a^

	Analyzed sample (n = 1072)	Excluded sample (n = 735)	*P* value^b^
Age (y)	35.6 (4.4)	36.0 (4.1)	.09
Treatment type^c^			
A (Cycle with fresh ET)	440 (41.0)	171 (23.3)	**<.001**
B (Cycle with freeze‐all and a subsequent FET)	445 (41.5)	383 (52.1)	
C (Frozen ET cycles)	20 (1.9)	24 (3.3)	
D (Cancelation due to patients’ health problem)	33 (3.1)	56 (7.6)	
E (Cancelation due to unfertilized egg/ embryo development)	116 (10.8)	94 (12.8)	
F (Cancelation due to non‐oocyte collected)	18 (1.7)	7 (0.95)	
Fertilization method			
IVF+ICSI	92 (8.6)	53 (7.2)	.59
IVF only	413(38.5)	260 (35.4)	
ICSI only	416 (38.8)	284 (38.6)	
Missing	151 (14.1)	138 (19.8)	
Housewife	419 (39.1)	273 (37.1)	.40
Total no of subsidies	2.34 (1.28)	2.55 (1.44)	**.002**
Annual couple's income (JPY)	4 321 475 (1 539 174)	4 509 354 (1 478 917)	**.01**

Abbreviations: ET, embryo transfer; FET, frozen‐thawed embryo transfer; ICSI, intracytoplasmic sperm injection; IVF, in vitro fertilization; JPY, Japanese Yen.

^a^ Data are presented as the mean (± SD) for continuous variables and n (%) for dichotomous variables.

^b^ Assessed using chi‐square or Student's *t* test.

^c^ Treatment type for first application.

Baseline characteristics for the analyzed sample stratified by the women's age are shown in Table 2. For the treatment type at first application, the proportion of freeze‐all and a subsequent FET cycles was higher in the younger age group, while the proportions of canceled cycles were higher in the older age group with statistical significance (*P* < .001). For infertility diagnosis, the proportions of tubal factor and male factor infertility diagnoses were significantly higher in the younger age group (*P* = .005 for tubal factor and *P* = .04 for male factor, respectively), while other infertility diagnoses were more prevalent in the older age group.

**TABLE 2:** Baseline characteristics for the analyzed sample stratified by age (n = 1072)^a^

	< 35 (n = 413)	35‐39 (n = 438)	40‐42 (n = 221)	*P* value
Age (y)	31.0 (2.6)	37.1 (1.4)	41.2 (1.3)	<.001^b^
Treatment type at first application^e^				
A (Cycle with fresh ET)	167 (40.4)	177 (40.4)	96 (43.4)	<.001^c^
B (Cycle with freeze‐all and a subsequent FET)	187 (45.3)	190 (43.4)	68 (30.8)	
C (Frozen ET cycles)	5 (1.2)	8 (1.8)	7 (3.2)	
D (Cancelation due to patients' health problem)	20 (4.8)	8 (1.8)	5 (2.3)	
E (Cancelation due to unfertilized egg/ embryo development)	31 (7.5)	47 (10.7)	38 (17.2)	
F (Cancelation due to non‐oocyte collected)	3 (0.73)	8 (1.8)	7 (3.2)	
Housewife	151 (36.6)	173 (39.5)	95 (43.0)	<.001^d^
Annual household income (JPY)	4 258 525 (1 506 548)	4 370 113 (1 535 059)	4 342 615 (1 609 110)	.80^b^
Quartile of annual couple's income				
Q1 (3,232,000 JPY)	105 (25.4)	107 (24.4)	56 (25.3)	.95^d^
Q2 (3 246 714‐4 389 754 JPY)	108 (26.2)	107 (24.4)	53 (24.0)	
Q3 (4 396 909‐5 466 400 JPY)	105 (25.4)	108 (24.7)	55 (24.9)	
Q4 (5 473 600‐7 292 085 JPY)	95 (23.0)	116 (26.5)	57 (25.8)	
Mean total no of subsidies	2.2 (1.2)	2.5 (1.5)	2.2 (0.79)	<.001^b^
Infertility diagnosis				
Tubal factor	74 (17.9)	52 (11.9)	21 (9.5)	**.005** ^d^
Endometriosis	21 (5.1)	22 (5.0)	9 (4.1)	.83^d^
Anti‐sperm antibody	2 (0.48)	0 (0)	0 (0)	.19^c^
Male factor	79 (19.1)	93 (21.2)	29 (13.1)	**.04** ^d^
Unexplained	238 (57.6)	260 (59.4)	147 (66.5)	.08^d^
Others	48 (11.6)	56 (12.8)	46 (20.8)	**.004** ^d^

Abbreviations: ET, embryo transfer; FET, frozen‐thawed embryo transfer; ICSI, intracytoplasmic sperm injection; IVF, in vitro fertilization.

^a^ Data are presented as mean (± SD) for continuous variables and n (%) for dichotomous variables.

^b^ Assessed using one‐way analysis of variance.

^c^ Assessed using Fisher's exact test.

^d^ Assessed using chi‐square test.

^e^ Treatment type for first application.

**FIGURE 2:**
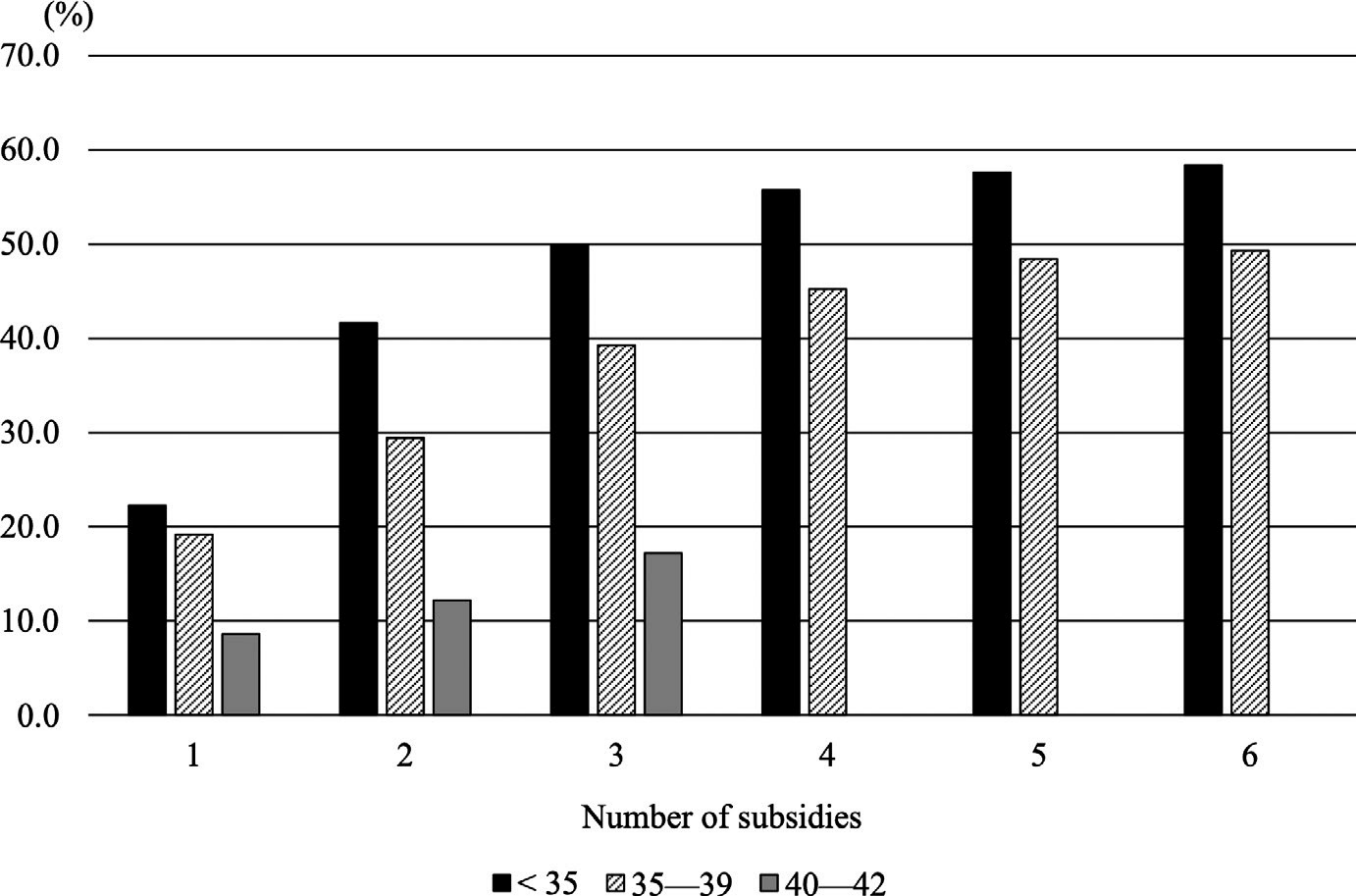
Cumulative live birth rate (CLBR) according to the numbers of applications stratified by women's age. For women aged 40‐42 y at the time of making the first application, only three subsidies were allowed

The CLBR according to the number of applications stratified by women's age is shown in Figure 2. Of 1,072 women included, 495 (46.2%) actually had a live birth, of which eight (1.6%) were twin pregnancies. 339 live births (68.5%) were derived from FET cycles, and there was no significant association between treatment type (A‐C) and patients’ age in live birth cases (*P* = .71). The CLBR over six ART subsidies was 53.7% for women aged <40 years and 17.2% over three subsidies for women aged 40‐42 years; it was highest among women aged <35 years (58.4%), followed by those in the 35‐39 years group (49.3%). The CLBR increased up to the fourth or fifth application but plateaued gradually between that and the sixth application. Detailed numbers of applicants and live births according to the number of governmental subsidies are shown in Table S1.

Treatment information for fresh cycles at the first application stratified by age group is shown in Table 3. For ovarian stimulation, natural cycles were selected for 14% of women across all age groups, while mild ovarian stimulation using clomiphene citrate (CC) alone was more often selected in the older group. GnRH agonist and antagonist protocols accounted for almost half of the cycles for women aged <35 years, but the proportions decreased with age. The mean number of oocytes collected was highest in those aged <35 years (mean 8.0, SD ± 7.2), while that in the 40‐42 years group was 4.2 (SD ± 5.2). Fresh ET was conducted in 44% to 48% of all age groups, and single embryo transfer (SET) was conducted in 98.8% of women aged <35 years, in 90.3% of those aged 35‐39 years, and in 80.0% of those aged 40‐42 (*P* < .001). The mean number of frozen embryos was the highest in those aged <35 years (mean 2.5, SD ± 3.1), while it was the lowest (mean 1.0, SD ± 1.7) in those aged 40‐42 years with statistical significance (*P* < .001).

**TABLE 3:** Treatment information for fresh cycles at first application stratified by age at the first application for ART subsidy (n = 980)^a^

	<35 (n = 379)	35‐39 (n = 398)	40‐42 (n = 203)	*P* value
Ovarian stimulation protocols				
Natural	53 (14.0)	57 (14.3)	29 (14.3)	**.01** ^b^
CC	31 (8.2)	67 (16.8)	44 (21.7)	
CC + Gn	63 (16.6)	61 (15.3)	34 (16.8)	
Gn only	9 (2.4)	4 (1.0)	4 (2.0)	
GnRH agonist	79 (20.8)	82 (20.6)	34 (16.8)	
GnRH antagonist	110 (29.0)	98 (24.6)	39 (19.2)	
Others	27 (7.1)	22 (5.5)	15 (7.4)	
Missing	7 (1.9)	7 (1.8)	4 (2.0)	
Cancelation cycles	0 (0)	0 (0)	2 (0.99)	**.04** ^c^
Number of oocytes collected	8.0 (7.2)	6.1 (5.5)	4.2 (5.2)	**<.001** ^d^
Women with no oocyte collected	3 (0.79)	6 (1.5)	6 (3.0)	**.13** ^c^

	n = 376	n = 392	n = 195	
Fertilization methods^e^				
IVF	162 (43.1)	153 (39.0)	78 (40.0)	.55^c^
ICSI	133 (35.4)	163 (41.6)	82 (42.1)	
IVF+ICSI	79 (21.0)	75 (19.1)	35 (18.0)	
Others	2 (0.53)	1 (0.26)	0 (0)	
Cycles with fresh ET^e^	166 (44.1)	176 (44.9)	94 (48.2)	.64^b^
Single ET rate^f^	164 (98.8)	159 (90.3)	75 (80.0)	<.001^b^
Cycle with freeze‐all^e^	176 (46.8)	164 (41.8)	61 (31.3)	**.002** ^b^
Number of frozen embryo^e^	2.5 (3.1)	1.8 (2.5)	1.0 (1.7)	**<.001** ^d^

Abbreviations: CC, clomiphene citrate; ET, embryo transfer; Gn, gonadotropin; ICSI, intracytoplasmic sperm injection; IVF, in vitro fertilization.

^a^ Data are presented as mean (SD) for continuous variables and n (%) for dichotomous variables.

^b^ Assessed using chi‐square test.

^c^ Assessed using Fisher's exact test.

^d^ Assessed using one‐way analysis of variance.

^e^ Women with canceled cycles and no oocytes collected were excluded.

^f^ The denominator was cycles with fresh ET.

The RRs and 95% CIs of patient characteristics for having a live birth are shown in Table 4. In the crude analysis, a diagnosis of male factor infertility was significantly positively associated with having a live birth (RR 1.17; 95% CI 1.003‐1.36). Similarly, compared with the lowest quartile of annual couple's income, the third highest quartile demonstrated a higher RR for having a live birth (RR 1.22; 95% CI 1.01‐1.47). However, age remained the only significant association for the having a live birth in the adjusted model. Compared with women aged <35 years, women aged 35‐39 and 40‐42 years demonstrated significantly decreased RRs (0.84; 95% CI 0.74‐0.96 and 0.30; 95% CI– 0.22‐0.40, respectively).

**TABLE 4:** Relative risks (RRs) and 95% confidence intervals (CIs) for having a live birth (n = 1072)

	Crude RR (95% CI)	Adjusted RR (95% CI)^a^
Age		
<35	Reference	Reference
35‐39	**0.85 (0.75‐0.96)**	**0.84 (0.74‐0.96)**
40‐42	**0.29 (0.22‐0.40)**	**0.30 (0.22‐0.40)**
Infertility diagnosis		
Tubal factor	0.97 (0.80‐1.17)	0.87 (0.69‐1.10)
Endometriosis	1.04 (0.78‐1.39)	0.99 (0.72‐1.34)
Anti‐sperm antibody	1.08 (0.27‐4.34)	0.82 (0.20‐3.36)
Male factor	**1.17 (1.003‐1.36)**	1.03 (0.83‐1.27)
Unexplained	0.93 (0.82‐1.06)	0.91 (0.73‐1.14)
Others	0.86 (0.70‐1.06)	0.94 (0.76‐1.16)
Quartile of annual couple's income		
Q1 (3,232,000 JPY)	Reference	Reference
Q2 (3,246,714‐4,389,754 JPY)	1.12 (0.92‐1.36)	1.11 (0.92‐1.33)
Q3 (4,396,909‐5,466,400 JPY)	**1.22 (1.01‐1.47)**	1.16 (0.97‐1.39)
Q4 (5,473,600‐7,292,085 JPY)	1.16 (0.96‐1.41)	1.15 (0.96‐1.38)
Housewife	0.88 (0.77‐1.01)	0.95 (0.83‐1.08)

Note

Significantly increased or reduced relative risks are indicated by boldface.

Abbreviation: RR, relative risk.

^a^ Adjusted for all variables listed.

## 4 DISCUSSION

In this retrospective study using individual data for women receiving governmental subsidies based on their first application in 2016, linked with the Japanese ART registry in which detailed treatment information was included, we found that the overall CLBR across six sets of subsidies for women aged <40 years was 53.7%; highest among women aged <35 years (58.4%), followed by those aged 35‐39 years (49.3%). For women aged 40‐42 years who were allowed three sets of subsidies, the CLBR was 17.2%. Multivariate analysis revealed only patient age as an independent factor associated with having a live birth. To date, this is the first study using individual treatment information to evaluate the CLBR according to the number of governmental subsidies for ART cycles in Japan.

Governmental subsidies for ART were first introduced in Japan in 2004 to reduce the economic burden for couples receiving infertility treatment and expanded step‐by‐step. At first, 100 000 JPY (960 USD) per fiscal years was provided for a 2‐year period for women whose maximum annual couple's income was 6 500 000 JPY (62,500 USD). From 2007, it was changed to 100,000 JPY per treatment cycle, and patients could apply for the subsidies twice per years for a 5‐year period. The upper limit of couple's income was also elevated to 7,300,000 JPY. The amount of subsidies per treatment cycle was raised to 150,000 JPY in 2009 and further increased to 300,000 JPY for first‐time applications since 2015. From January 2021, patients can now receive up to 300,000 JPY per treatment for fresh cycles per treatment for six attempts, and the limitation on annual couple's income has been discontinued.^7^

14% of patients selected natural cycle IVF for ovarian stimulation at first application, and mild ovarian stimulation using CC accounted for more than 20% across all age groups (Table 3). This indicates the Japanese typical tendency to favor mild ovarian stimulation. Also, freeze‐all cycles accounted for more than 46.8% in age <35 years. Among 401 cycles of freeze‐all cycle in which ovarian stimulation protocols were available at first application, 120 (29.9%) were GnRH antagonist protocols followed by 111 (27.7%) of GnRH agonist protocol, 14.0% of CC +gonadotropin, and 11.2% of CC alone. Thus, we speculated that a freeze‐all strategy with a subsequent FET was mainly used for strong ovarian stimulation to avoid risks for ovarian hyperstimulation syndrome (OHSS). Although ART facilities mainly conducting mild ovarian stimulation is rare in Saitama prefecture, the reason for selecting ovarian stimulation using natural cycle or CC even in younger patients is that not a small proportion of patients might receive ART treatment mainly conducting mild ovarian stimulation in other prefectures such as Tokyo.

With the expansion of governmental subsidies for ART, the number of ART treatment cycles in Japan has been increasing continuously. According to the latest report from the JSOG, 454,893 treatment cycles including IVF, ICSI, and FET were conducted in 2018, and 56,979 neonates were born from ART.^1^ The number of treatment cycles itself has been very high in terms of international comparisons; from the latest International Committee for Monitoring Assisted Reproductive Technology (ICMART) preliminary report, Japan was the second largest absolute user of ART in the world in 2016.^10^ The ART utilization rate, expressed as the number of cycles per million population per annum, is an effective indicator of access to infertility care,^11^ and Japan had the highest worldwide (3,212 cycles/million), which was ~500 times higher than Senegal, the country with the lowest rate (6 cycles/million). Importantly, the mean age of women receiving ART was extremely high in Japan: The proportion of registered cycles applied to women aged ≥40 years was 41.8% in 2018. This compares with the following other developed countries: 28.3% in Australia, 22.1% in Germany, and 23.9% in the United States according to the ICMART report.^5^ Because ART using donor oocytes/embryos is almost never practiced in Japan, older women have to continue treatment multiple times to achieve a live birth compared with younger women, so advanced patient age has been the major reason for the increased numbers of ART treatment cycles in Japan.

To improve the live birth impact of governmental subsidies for ART, age limitations for subsidies and incentives for younger women were introduced in 2016; subsidies were only available for women aged <43 years, and patients aged 40‐42 y can only apply for three attempts, while patients aged <40 years can apply for six attempts. In fact, the CLBR in our analysis was the highest in women aged <35 years (58.4%). Considering that patient age was the only significant background factor for having a live birth in our study, incentives for younger couples would be a reasonable strategy for effective subsidies.

One of the other clinical outcomes in this study was a multiple pregnancy rate of only 1.6% (eight sets of twins), which is exceptionally low compared with other developed countries.^5^ This low prevalence is largely attributed to the high SET rate of 82%–83% in 2018 ^12^ in Japan following JSOG recommendations in 2008.^13^ Moreover, the high accessibility of Japanese to ART treatment might also have played a role. It has been hypothesized that countries with restricted accessibility to ART, such as those with underinsurance or very limited health insurance coverage, tend to choose aggressive treatments such as ovarian hyperstimulation and multiple embryo transfer,^14^ which resulted in very high multiple pregnancy rates.^15^ In Japan, the ease of access to ART treatment might help decrease the demand of patients for more aggressive treatments. To reduce the high burden of healthcare costs of caring for multiple‐birth infants after ART as well as maternal risks for pregnancy complications,^16^, ^17^, ^18^ several countries (eg, Belgium, New Zealand, and Turkey) and states (eg, Quebec in Canada and Connecticut in the United States) have introduced health insurance coverage for ART linked to SET policy, and have successfully achieved significant reductions both in multiple pregnancy rates and in related healthcare costs.^19^, ^20^, ^21^, ^22^

In this study, nearly 60% of younger patients (<35 years) had a live birth after at least 1 year. To date, evaluation of the Japanese governmental subsidies, in terms of clinical practice, and outcomes, such as the CLBR, have not been fully investigated. Because the Japanese ART registry only includes cycle‐specific information and cannot distinguish between individual patients receiving multiple treatment, only live birth rates per total number of treatment cycles with or without using subsidies were available. Thus, the CLBR using our data would be informative for infertile couples to decide on the implementation of ART using governmental subsidies.

This is the first study to investigate the impact of governmental subsidies for ART on the CLBR in Japan using individual data for governmental ART subsidies linked with the Japanese national ART registry. However, there were several limitations. First, we linked individual data with the Japanese ART registry using unique IDs for treatment cycles, but a significant proportion of these (39.0%) lacked this information. This might have introduced selection bias for the sample selected. Characteristics based on application information stratified by included and excluded status demonstrated that treatment type, the mean total number of subsidies, and mean annual couple's income were significantly different between the included and excluded samples (Table 1). In particular, women who applied multiple times for subsidies tended to be excluded, and this would have overestimated the CLBR in our analysis. Similarly, couples with an annual income >7,300,000 JPY were excluded because of the income limit for governmental subsidies, and the CLBR for those couples is unknown. Thus, evaluation of the effectiveness of governmental subsidies is very difficult. Further, the study sample covered by the Saitama Prefectural Government does not necessarily represent Japan, and different results might be obtained in a different area of Japan. In this study, major cities in Saitama prefecture were excluded, in which 12 (44.4%) out of 27 ART facilities exist. Although it is not possible to know the actual number of ART facilities included in the analyzed data, the representativeness of the study results is unknown. Second, the study only targeted women making their first application in 2016 who were followed up retrospectively to the end of 2017. This follow‐up period might not have been adequate. Longer follow‐ups, especially for women aged <40 years, are necessary to increase the proportion of women who complete the total number of available subsidies in the future. Further, for women who have surplus frozen embryos but no more chance for subsidies, live births from the embryos were not counted in this study due to the methodological limitation. Third, although we linked the data with the Japanese ART registry, the study still lacked important confounding information such as any history of previous children arising from ART treatment,^23^ body mass index,^24^ and duration of infertility.^25^ These unmeasured confounders would invariably affect the multivariable analysis.

Although the Japanese government has recently indicated it plans to cover infertility treatment by social health insurance by 2022, the establishment of sustainable healthcare systems is essential. For example, in Quebec, Canada, it was widely reported that the abovementioned universal coverage of IVF in conjunction with a SET policy meant that the access to IVF was dramatically improved,^26^ which resulted in a significant decline in the multiple pregnancy rate.^22^ However, in 2015, the system was dismantled because of costs to the healthcare system.^19^ Thus, for a sustainable system, clear indications and appropriate restrictions for the use of ART under health insurance coverage are necessary. In addition, continuous monitoring of ART utilization and outcome measurements will be indispensable.

## 5 CONCLUSIONS

In conclusion, this study, using individual data for governmental ART subsidies and linked with the Japanese national ART registry, demonstrated that the overall CLBR across six subsidies was 53.7%, in which women aged <35 years had the highest rate (58.4%), while 49.3% of the women aged 35‐39 years actually had a live birth. For women aged 40‐42 years for whom only three subsidies were allowed, the CLBR was 17.2%. Given that age was the only significant factor associated with CLBR, policies for promoting ART among younger couples who seek infertility treatment in Japan are essential.

## CONFLICT OF INTEREST

Dr Osamu Ishihara has received an honorarium from Ferring Pharmaceuticals.

## ETHICS APPROVAL

This study was approved by the institutional review board of Saitama Medical University (Approval number, 904; September 2019) and the ethics committee of the JSOG (Approval number 2020‐2; June 2020).

## HUMAN RIGHTS STATEMENT AND INFORMED CONSENT

All procedures were performed in accordance with the ethical standards of the relevant committees on human experimentation (institutional and national) and the Helsinki Declaration of 1964 and its later amendments.

## ANIMAL RIGHTS

This report does not contain any studies performed by any of the authors that included animal participants.

## Supporting information

Table S1

## References

[1] Ishihara O , et al. Assisted reproductive technology in Japan: A summary report for 2018 by the Ethics Committee of the Japan Society of Obstetrics and Gynecology. Reprod Med Biol. 2021;20(1):3–12. 33488278 10.1002/rmb2.12358 PMC7812461

[2] Ministry of Health Labour and Welfare . Summary of vital statistics in 2018 (In Japanese). Tokyo. 2019. https://www.mhlw.go.jp/toukei/saikin/hw/jinkou/kakutei18/dl/02_kek.pdf Accessed: 13/Sep/2020

[3] Baird DT , et al. Fertility and ageing. Hum Reprod Update. 2005;11(3):261‐276. 15831503 10.1093/humupd/dmi006

[4] Cabinet Office . Declining Birthrate White Paper (In Japanese). Tokyo. 2020. https://www8.cao.go.jp/shoushi/shoushika/whitepaper/measures/w‐2020/r02pdfhonpen/r02honpen.html Accessed: 17/Jan/2021

[5] de Mouzon J , et al. International Committee for Monitoring Assisted Reproductive Technologies world report: assisted reproductive technology 2012dagger. Hum Reprod. 2020;35:1900‐1913. 32699900 10.1093/humrep/deaa090

[6] Ikegami N , et al. Japanese universal health coverage: evolution, achievements, and challenges. Lancet. 2011;378(9796):1106‐1115. 21885107 10.1016/S0140-6736(11)60828-3

[7] Ministry of Health, Labour and Welfare . Enlargement of subsidies for assisted reproductive technology (In Japanese). Tokyo. 2020. https://www.mhlw.go.jp/stf/seisakunitsuite/bunya/0000047270.html. Accessed: 17/Jan/2021.

[8] Working group for appropriate governmental subsidies for infertility patients. Report of appropriate governmental subsidies for infertility patients (In Japanese). 2013. https://www.mhlw.go.jp/file/04‐Houdouhappyou‐11908000‐Koyoukintoujidoukateikyoku‐Boshihokenka/0000016937.pdf Accessed: 17/Jan/2021

[9] Greenland S . Model‐based estimation of relative risks and other epidemiologic measures in studies of common outcomes and in case‐control studies. Am J Epidemiol. 2004;160:301‐305. 15286014 10.1093/aje/kwh221

[10] Adamson GD , et al. International Committee for Monitoring Assisted Reproductive Technology: world report on assisted reproductive technology, 2016. 2020. https://secureservercdn.net/198.71.233.206/3nz.654.myftpupload.com/wp‐content/uploads/ICMART‐ESHRE‐WR2016‐FINAL‐20200901.pdf Accessed: 13/Sep/2020

[11] Dyer S , Chambers GM , Adamson GD , et al. ART utilization: an indicator of access to infertility care. Reprod Biomed Online. 2020;41(1):6‐9. 32448672 10.1016/j.rbmo.2020.03.007

[12] Ishihara O , Jwa SC , Kuwahara A , et al. Assisted reproductive technology in Japan: a summary report for 2017 by the Ethics Committee of the Japan Society of Obstetrics and Gynecology. Reprod Med Biol. 2020;19:3‐12. 31956280 10.1002/rmb2.12307 PMC6955594

[13] Takeshima K , Jwa SC , Saito H , et al. Impact of single embryo transfer policy on perinatal outcomes in fresh and frozen cycles‐analysis of the Japanese Assisted Reproduction Technology registry between 2007 and 2012. Fertil Steril. 2016;105(337–46):e3. 10.1016/j.fertnstert.2015.10.002 26518122

[14] Johnston J , et al. Preterm births, multiples, and fertility treatment: recommendations for changes to policy and clinical practices. Fertil Steril. 2014;102:36‐39. 24739454 10.1016/j.fertnstert.2014.03.019

[15] Chambers GM , Keller E , Choi S , et al. Funding and public reporting strategies for reducing multiple pregnancy from fertility treatments. Fertil Steril. 2020;114:715‐721. 33040980 10.1016/j.fertnstert.2020.08.1405

[16] Chambers GM , et al. The economic implications of multiple pregnancy following ART. Semin Fetal Neonatal Med. 2014;19(4):254‐261. 24933114 10.1016/j.siny.2014.04.004

[17] Chambers GM , et al. Hospital costs of multiple‐birth and singleton‐birth children during the first 5 years of life and the role of assisted reproductive technology. JAMA Pediatr. 2014;168(11):1045‐1053. 25222633 10.1001/jamapediatrics.2014.1357

[18] Santana DS , Cecatti J , Surita FG et al. Twin pregnancy and severe maternal outcomes: The World Health Organization Multicountry Survey on Maternal and Newborn Health. Obstet Gynecol. 2016;127:631‐641. 26959199 10.1097/AOG.0000000000001338

[19] Wei SQ , Bilodeau‐Bertrand M , Lo E , Auger N . Effect of publicly funded assisted reproductive technology on maternal and infant outcomes: a pre‐ and post‐comparison study. Hum Reprod. 2021;36:219‐228. 33246340 10.1093/humrep/deaa270

[20] Peeraer K , Debrock S , Laenen A , et al. The impact of legally restricted embryo transfer and reimbursement policy on cumulative delivery rate after treatment with assisted reproduction technology. Hum Reprod. 2014;29:267‐275. 24282120 10.1093/humrep/det405

[21] Kutlu P , Atvar O , Vanlioglu OF , et al. Effect of the new legislation and single‐embryo transfer policy in Turkey on assisted reproduction outcomes: preliminary results. Reprod Biomed Online. 2011;22(2):208‐214. 21195669 10.1016/j.rbmo.2010.10.007

[22] Velez MP , et al. Universal coverage of IVF pays off. Hum Reprod. 2014;29:1313‐1319. 24706002 10.1093/humrep/deu067

[23] Paul RC , et al. Cumulative live birth rates for women returning to ART treatment for a second ART‐conceived child. Hum Reprod. 2020;35:1432‐1440. 32380547 10.1093/humrep/deaa030

[24] Goldman RH , et al. The combined impact of maternal age and body mass index on cumulative live birth following in vitro fertilization. Am J Obstet Gynecol. 2019;221(6):e1‐e13. 10.1016/j.ajog.2019.05.043 31163133

[25] McLernon DJ , et al. Predicting the chances of a live birth after one or more complete cycles of in vitro fertilisation: population based study of linked cycle data from 113 873 women. BMJ. 2016;355: i5735. 27852632 10.1136/bmj.i5735 PMC5112178

[26] Tulandi T , King L , Zelkowitz P . Public funding of and access to in vitro fertilization. N Engl J Med. 2013;368:1948‐1949. 23675672 10.1056/NEJMc1213687

