# Supplementary material for: Cumulative live birth rate according to the number of receiving governmental subsidies for assisted reproductive technology in Saitama Prefecture, Japan: A retrospective study using individual data for governmental subsidies
Source: Reprod Med Biol. 2021 Jun 20;20(4):451–9. doi: 10.1002/rmb2.12397 (PMC8499586; doi:10.1002/rmb2.12397)
Supplement: Supplementary file 1 — Table S1 [file RMB2-20-451-s001.docx]

**Supporting Information**

**Table S1** Number of applicants and live births according to the number of governmental subsides stratified by age.

| Table S1. Number of applicants and live births according to the number of governmental subsides stratified by age. | | | | |
| --- | --- | --- | --- | --- |
| Number of governmental subsidies | Number of applicants | Number of live births | Cumulative number of live births | CLBR (%) |
| **Age <35** |  |  |  |  |
| 1 | 413 | 92 | 92 | 22.3 |
| 2 | 264 | 80 | 172 | 41.6 |
| 3 | 139 | 34 | 206 | 49.9 |
| 4 | 69 | 24 | 230 | 55.7 |
| 5 | 25 | 8 | 238 | 57.6 |
| 6 | 6 | 3 | 241 | 58.4 |
| **Age 35–39** |  |  |  |  |
| 1 | 438 | 84 | 84 | 19.2 |
| 2 | 287 | 45 | 129 | 29.5 |
| 3 | 198 | 43 | 172 | 39.3 |
| 4 | 109 | 26 | 198 | 45.2 |
| 5 | 51 | 14 | 212 | 48.4 |
| 6 | 18 | 4 | 216 | 49.3 |
| **Age 40–42** |  |  |  |  |
| 1 | 221 | 19 | 19 | 8.6 |
| 2 | 168 | 8 | 27 | 12.2 |
| 3 | 88 | 11 | 38 | 17.2 |
| CLBR, cumulative live birth rate. | |  |  |  |
